# Antagonistic L1 Adhesion Molecule Mimetic Compounds Inhibit Glioblastoma Cell Migration In Vitro

**DOI:** 10.3390/biom12030439

**Published:** 2022-03-12

**Authors:** Vini Nagaraj, Mirai Mikhail, Micol Baronio, Alessia Gatto, Ashana Nayak, Thomas Theis, Ugo Cavallaro, Melitta Schachner

**Affiliations:** 1 Keck Center for Collaborative Neuroscience, Department of Cell Biology and Neuroscience, Rutgers, The State University of New Jersey, Piscataway, NJ 08854, USA; vn149@dls.rutgers.edu (V.N.); miraimikhail@gmail.com (M.M.); asn77@scarletmail.rutgers.edu (A.N.); theis@dls.rutgers.edu (T.T.); 2 Unit of Gynaecological Oncology Research, European Institute of Oncology IRCSS, 20139 Milan, Italy; micol.baronio@ieo.it (M.B.); alessia.gatto@ieo.it (A.G.); ugo.cavallaro@ieo.it (U.C.)

**Keywords:** L1CAM, CD171, small compound libraries, monoclonal L1 antibody 324, antagonist mimetics, migration, tumor progression

## Abstract

Cell adhesion molecule L1 is a cell surface glycoprotein that promotes neuronal cell migration, fosters regeneration after spinal cord injury and ameliorates the consequences of neuronal degeneration in mouse and zebrafish models. Counter-indicative features of L1 were found in tumor progression: the more L1 is expressed, the more tumor cells migrate and increase their metastatic potential. L1′s metastatic potential is further evidenced by its promotion of epithelial–mesenchymal transition, endothelial cell transcytosis and resistance to chemo- and radiotherapy. These unfortunate features are indicated by observations that cells that normally do not express L1 are induced to express it when becoming malignant. With the aim to ameliorate the devastating functions of L1 in tumors, we designed an alternative approach to counteract tumor cell migration. Libraries of small organic compounds were screened using the ELISA competition approach similar to the one that we used for identifying L1 agonistic mimetics. Whereas in the former approach, a function-triggering monoclonal antibody was used for screening libraries, we here used the function-inhibiting monoclonal antibody 324 that reduces the migration of neurons. We now show that the L1 antagonistic mimetics anagrelide, 2-hydroxy-5-fluoropyrimidine and mestranol inhibit the migration of cultured tumor cells in an L1-dependent manner, raising hopes for therapy.

## 1. Introduction

The cell adhesion molecule L1, also called L1CAM or CD171, was the founding member of the L1 family of adhesion molecules, a subgroup of the immunoglobulin superfamily of adhesion molecules which display overlapping but also distinct functions (for a recent review, see [1]). L1 was the first cell surface glycoprotein shown to be involved in mammalian neuronal cell migration using monoclonal and polyclonal antibodies [2,3]. After its initial characterization, many more functions were shown to depend on L1, including neuronal survival, axonal outgrowth, guidance and fasciculation, synapse formation and synaptic plasticity. Homophilic, i.e., self-binding, and non-self-binding heterophilic interactions revealed several binding partners that cooperate with L1 in regulating neurite outgrowth and neural cell networking [4,5]. In addition to contributing to the development of the central and peripheral nervous systems, L1 was discovered to mediate regeneration after injury in several injury model paradigms, as exemplified by L1-induced axonal regrowth and remyelination after injuries to rodent and zebrafish spinal cords and mouse peripheral nerves [6,7,8]. Indeed, when applied in spinal cord injury models as a recombinant glycoprotein or when overexpressed by adeno-associated virus in stem cell-derived neural aggregates, Schwann cells and radial glial cells, L1 accelerated remyelination and improved axonal regrowth/sprouting/sparing proximal, distal and across the lesion site [9,10,11,12]. In addition, L1 ameliorated the severe consequences of injury in experimental models of neurodegenerative diseases in vitro and in vivo [13,14,15,16,17]. Since the viral delivery of L1, application of recombinant L1, and injection of stem cells overexpressing L1 are expected to meet difficulties in translation to therapy, libraries of small organic molecules were screened for compounds that structurally and functionally mimic L1, and several molecules were found to act as L1 agonistic mimetics [18]. These L1 mimetic agonists were shown to support recovery after injury not only in mice but also in zebrafish [19,20].

An increasing body of clinical and experimental evidence has established a causal role for L1. A counter-indicative feature of L1 was found in tumor progression. The more L1 is expressed, the more tumor cells migrate to increase their metastatic potential [21]. L1’s metastatic potential is further evidenced by its presence on pericytes [22], promotion of epithelial–mesenchymal transition, endothelial cell transcytosis [23] and rendering different types of tumors resistant to therapy [24]. This unfortunate feature of L1 is indicated by the observation that cells that normally do not express L1 are induced to express it when becoming malignant [25].

The aberrant expression of L1 in different cancer types correlates frequently with a poor outcome [24]. Such a correlation is tightly linked to the ability of L1 to enhance tumor cell proliferation, invasion, and metastatic dissemination [25,26]. Interesting in this context is the report that the aspirated cyst fluid in glioblastoma cells and brain system tumors is increased [27,28]. The role of L1 in the transendothelial migration of cancer cells [23,29] likely contributes to their metastatic potential. Along this line, L1 has been recently described as a marker and a driver in metastasis-initiating cells [22,30]. Finally, L1 has been causally implicated in cancer stemness and in chemoresistance (reviewed in [24]). Taken together, these functional properties of L1 in the context of cancer progression imply that the inactivation of this protein might prove an efficacious anti-tumor strategy. Indeed, L1-targeted treatments based, for example, on neutralizing antibodies, radioimmunoconjugates or chimeric antigen receptor-redirected T (CAR-T) cells have given promising results in preclinical tumor models [24]. 

With the aim to minimize the devastating functions of L1 in tumors, we designed a novel approach to counteract tumor migration. Libraries of small organic compounds were screened using the ELISA competition approach similar to the one that we had used for identifying the agonistic L1 mimetics [18]. Whereas in the former approach, a function-triggering monoclonal antibody was used for screening in the competition ELISA, we here used a function-inhibiting monoclonal antibody that reduced the migration of developing, post-mitotic neurons, findings which led to the identification of the L1 glycoprotein. Here, we show that antagonistic L1 mimetics could be identified and that the migration of tumor cells can be inhibited in vitro in an L1-dependent manner. 

## 2. Materials and Methods

### 2.1. Antibodies and Reagents

Media and reagents for cell culture were from Gibco. The L1 mimetic compounds 6,7-dichloro-1,5-dihydroimidazo (2,1-b) quinazolin-2(3H)-one (anagrelide; CAS 68475-42-3), 5-fluoro-1H-pyrimidin-2-one (2-hydroxy-5-fluoropyrimidine; CAS 2022-78-8), and (8R,9S,13S,14S,17R)-17-ethynyl-3-methoxy-13-methyl-7,8,9,11,12,14,15,16-octahydro-6H-cyclopenta[a]phenanthren-17-ol (mestranol; CAS 72-33-3) were from Sigma-Aldrich (St. Louis, MO, USA). Ortho-phenylenediamine dihydrochloride (OPD, CAS 615-28-1), calcein-AM (CAS 148504-34-1), and propidium iodide (CAS 25535-16-4) were from Thermo Fisher Scientific (Waltham, MA, USA). The NIH Clinical Collection libraries 1 and 2 were from Evotec (San Francisco, CA, USA) and the Natural Product Library was from Selleckchem (Houston, TX, USA). The rat anti-mouse L1 monoclonal antibody 324 (CAS MAB5272) was from Sigma-Aldrich, and secondary donkey anti-rat IgG antibodies coupled to horseradish peroxidase (HRP) were from Jackson ImmunoResearch (West Grove, PA, USA). Recombinant mouse L1CAM-Fc (L1-Fc) chimera (CAS 5674-NC-050) was from R&D Systems (Minneapolis, MN, USA). Human IgG1-Fc tag free protein (CAS FCC-H5214) was from ACROBiosystems (Newark, DE, USA). L1CAM (clone UJ127; catalog# sc-533386) was from Santa Cruz (Dallas, TX, USA). GAPDH (Cat# 60004-1-Ig was from Proteintech (Rosemont, IL, USA)).

### 2.2. ELISA Screening and Verification of Mimetics

Low-throughput ELISA screening and verification of mimetics was performed as described [31], with modifications. Briefly, to identify the compounds that inhibit the binding of antibody 324 to mouse L1-Fc, NIH Clinical Collection 1 and 2 Libraries and the Natural Product Library were screened via competition ELISA. Mouse L1-Fc (2.5 μg/mL; 25 μL/well) was substrate-coated and wells were treated with blocking solution as described. In parallel, the compounds were incubated (40 μM) with 1 μg/mL antibody 324 for 30 min at room temperature. The compound–antibody solution was then transferred to the L1-Fc-coated plate (25 μL/well) and allowed to incubate for another 30 min under shaking. Detection of the antibody that had bound to the immobilized L1-Fc was measured as described under Supplementary ELISA with L1-Fc. To analyze the concentration dependence of this inhibition, the competition ELISA was repeated with some modifications: After blocking, the hit compounds were individually added to the wells at different concentrations (0, 10, 20, 60, 100, and 200 μM) and incubated on the shaker for 30 min. The compounds were then discarded and antibody 324 (1 μg/mL; 25 μL/well) was added to the wells and incubated on the shaker for 30 min. The antibody was then discarded, and the wells were washed three times with PBS. All following steps were repeated as described under Supplementary ELISA with L1-Fc. 

### 2.3. Cell Culture

U251 human glioblastoma cells (Sigma cat# 09063001-1VL) were cultured in Dulbecco’s Modified Eagle Medium (DMEM, Cat# 11995065; ThermoFisher Scientific, Waltham, MA, USA) supplemented with 10% fetal bovine serum (FBS, Cat# RLBSA50; VWR International (Radnor, PA, USA)), 1% MEM non-essential amino acids, 1% penicillin-streptomycin, 1% sodium pyruvate, and 1% glutaMAX. The human ovarian cancer cells (OVCAR3) were received from Ugo Cavallaro, as purchased from the American Type Culture Collection (ATCC) and cultured in RPMI 1640 (Cat# 11875093; ThermoFisher Scientific) supplemented with 20% FBS, 1% penicillin-streptomycin, 10 μg/mL bovine insulin and 1 μg/mL puromycin. The human embryonic kidney cell line HEK293T was purchased from ATCC and maintained in DMEM containing 10% FBS, 2 mM L-glutamine, 100 U/ ml penicillin, and 100 μg/mL streptomycin. All cells were maintained at 37 °C in 5% CO_2_.

### 2.4. Lentivirus Production and Cell Transduction

HEK293T was used as the packaging cell line for lentiviral particle production using the calcium phosphate precipitation method. A measure of 10 μg of lentiviral expression plasmids was co-transfected with 3 μg PMD2G, 5 μg RRE and 2.5 μg REV packaging plasmids and 61 µL 2M CaCl_2_. After a 48 h incubation, the supernatant was used to transduce the target cells U251 and OVCAR3 by the addition of 8 μg/mL of polybrene as an adjuvant to increase the transduction efficiency. U251 and OVCAR3 were transduced with lentiviral vectors containing either a scrambled shRNA (Catalog# CSHCTR001-LVRU6P) or the short-hairpin RNA sequences SH1 (Catalog# HSH010390—1-LVRU6P; ggatggtgtccacttcaaa), SH3 (Catalog# HSH010390–3-LVRU6P; ccaccaacagcatgattga), with all plasmids purchased from GeneCopoeia (Rockville, MD, USA). The U251-scrambled, U251-SH1, U251-SH3, OVCAR3-scrambled and OVCAR3-SH1 cell lines were then generated upon selection with 1 μg/mL (U251) and 2 μg/mL (OVCAR3) puromycin. Both cells were stably transduced and subjected to puromycin selection for 48–72 h. Cells were then cultured in the presence of puromycin and monitored regularly for the maintenance of L1 silencing. The migration experiments were all performed within three passages from the initial puromycin selection. Reduced expression of L1 in OVCAR3 cells silenced with SH1 and SH3 shRNA has recently been reported [32].

### 2.5. Calcein-AM/Propidium Iodide Toxicity Assay

U251 cells were plated (5 × 10^4^ cells/mL, 100 μL/well) in duplicates in 96-well plates and allowed to settle before treatment for 24 h with 0, 1, 10 and 100 μM anagrelide, 2-hydroxy-5-fluoropyrimidine or mestranol in vehicle solution (final concentration of DMSO in culture was 1%), after which 0.5 μL of a 1:1 solution containing 1 mg/mL calcein-AM and 1 mg/mL propidium iodide was added to each well. Cells were then incubated for 20 min at 37 °C and imaged immediately afterwards. Imaging of live cells was performed using a Zeiss Axiovert 200M inverted transmission-light microscope (Carl Zeiss, Oberkochen, Germany) with a 20× objective, aperture 0.3, and AxioVision 4.6 Software (Carl Zeiss, NY, USA).

### 2.6. Migration Assay

U251 (7.5 × 10^4^ cells/mL, 100 μL/well), U251-scrambled, U251-SH1, U251-SH3, OVCAR3-scrambled and OVCAR3-SH1 (1 × 10^5^ cells/mL, 100 μL/well) cells were plated in duplicates into 96-well plates and incubated for 24 h until confluent. A wound was then induced by scratching the monolayer with a 200 μL plastic pipette tip. The medium was then changed to medium containing 2% FBS (with all other medium components as described above under cell culture). The cells were then imaged immediately to determine the 0 h time point. After imaging, cells were treated with different concentrations of anagrelide, 2-hydroxy-5-fluoropyrimidine, or mestranol (in vehicle control, 1, 10 and 100 μM) and incubated for 24 h. The migration of cells was monitored by imaging the gap width every 24 h for 96 h. Live imaging of cells was performed using a Zeiss Axiovert 200M inverted transmission-light microscope (Carl Zeiss) with a 10× objective, aperture 0.25, and AxioVision 4.6 software. The gap width was quantified using ImageJ software. 

### 2.7. Stastistical Analysis

Average values and standard error of the mean (SEM) were calculated from 3 independent experiments, unless otherwise stated in the figure legends. Statistical comparisons between groups were performed by one-way analysis of variance (ANOVA) using Fisher’s protected least significant difference (PLSD) test, with StatView Version 5.0.1 (SAS Institute Inc., New York, NY, USA).

## 3. Results

### 3.1. Mimetics Competitively Inhibit Antibody 324 Binding to L1

For an initial competitive ELISA screen, we pre-incubated small organic compounds from the NIH Clinical Collection 1 and 2 Libraries and the Natural Product Library with antibody 324 before adding the antibody–compound mixtures to L1-Fc substrate-coated in 96-well plates. With this initial screen, we identified anagrelide, 2-hydroxy-5-fluoropyrimidine, and mestranol as small organic compounds that interfere with antibody binding to L1-Fc. In the presence of these three compounds, antibody binding to L1 was inhibited by at least 50% compared to control. 

**Figure 1:**
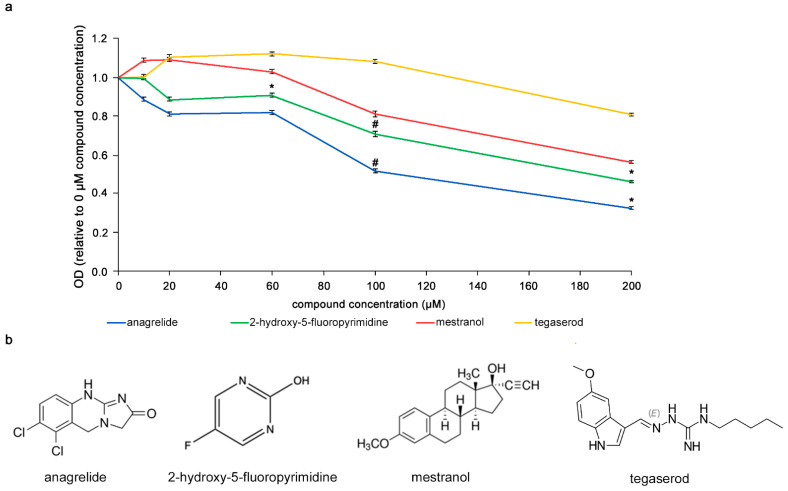
Competition ELISA with mimetics. (**a**) Concentration-dependent reduction of antibody 324 binding to substrate-coated L1-Fc by anagrelide, 2-hydroxy-5-fluoropyrimidine and mestranol. Tegaserod served as negative control. Compounds were incubated with antibody 324 and then tested for binding to substrate-coated mouse L1-Fc. Antibody binding to L1-Fc without compounds was set to 1 for absorbance/OD. Significance of differences between mimetics and tegaserod was determined by one-way ANOVA with Tukey’s post hoc test. * *p* < 0.05, # *p* < 0.01. The small error bars show mean ± SEM. (**b**) Chemical structure of mimetics and tegaserod.

It was investigated whether the compounds would bind to L1, i.e., in a homophilic binding mode, different concentrations (0–200 µM) of the compounds were incubated with L1-Fc substrate-coated in 384-well plates. The compounds were then removed and antibody 324 was added to the wells to allow binding to L1-Fc. Higher concentrations of anagrelide, 2-hydroxy-5-fluoropyrimidine and mestranol showed greater inhibition of antibody 324 binding (Figure 1). At 200 µM, the optical density (OD) ratio of anagrelide and 2-hydroxy-5-fluoropyrimidine was approximately 0.5 and 0.4, respectively, relative to the antibody-only control (0 µM compounds). These observations indicated that anagrelide and 2-hydroxy-5-fluoropyrimidine inhibit the binding of antibody 324 at this concentration. Moreover, both the compounds showed inhibition at 100 µM concentration compared to the control compound, tegaserod, a mimetic of polysialic acid [33] that is a small organic compound similar to the L1 mimetics and was therefore used as a negative control. We emphasize that mestranol showed only a tendency for inhibition at all concentrations tested. These results show that the mimetic compounds bind to mouse L1 in a homophilic manner and competitively reduce binding of antibody 324 to L1-Fc in a concentration-dependent manner. 

### 3.2. Mimetics Are Not Toxic to U251 Cells

**Figure 2:**
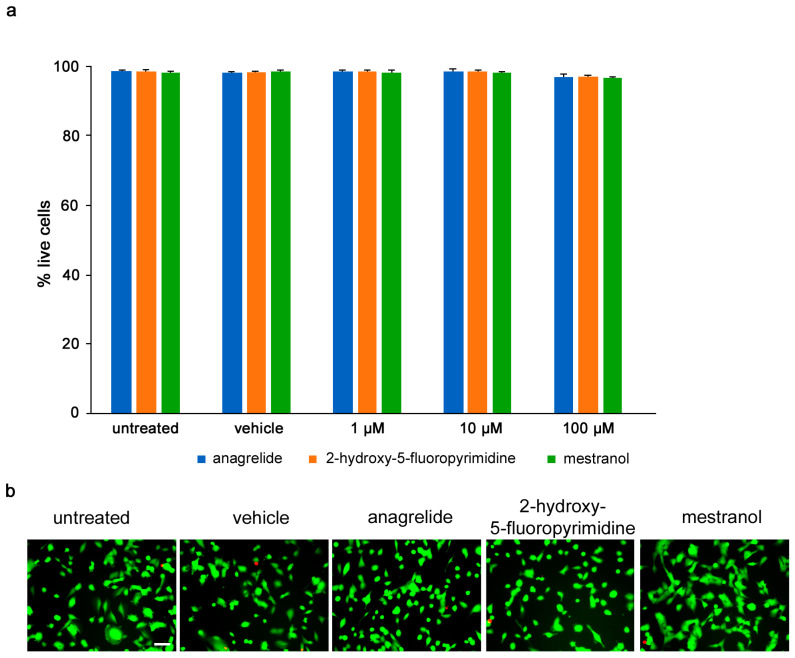
Mimetics are not toxic to U251 cells up to 100 µM. U251 cells were seeded at 5 × 10^4^ cells/mL, 100 μL/well in duplicates in 96-well plates. (**a**) Graph shows percentage of viable cells treated for 24 h with different concentrations of anagrelide, 2-hydroxy-5-fluoropyramidine or mestranol (1, 10, 100 μM) and then stained with calcein-AM and propidium iodide. Five images were taken per well, and values for each condition were averaged. Error bars show mean + SEM. (**b**) Representative images of untreated cells, vehicle-treated cells and cells treated with 100 μM mimetics. Green cells stained with calcein-AM indicate live cells and red nuclei stained with propidium iodide indicate dead cells. Scale bar for all images, 50 μm in panel for untreated cells. There was no difference between untreated cells and vehicle-treated cells.

Toxicity of compounds was determined by calcein-AM/propidium iodide stain, and the numbers of live and dead U251 cells were measured following treatment with different concentrations (1–100 µM) of each compound. Anagrelide, 2-hydroxy-5-fluoropyrimidine and mestranol all caused less than 5% of all cells to die at all concentrations tested and showed no difference between the untreated cells and vehicle control-treated cells (Figure 2).

### 3.3. Mimetics Inhibit Migration of U251 Cells

**Figure 3:**
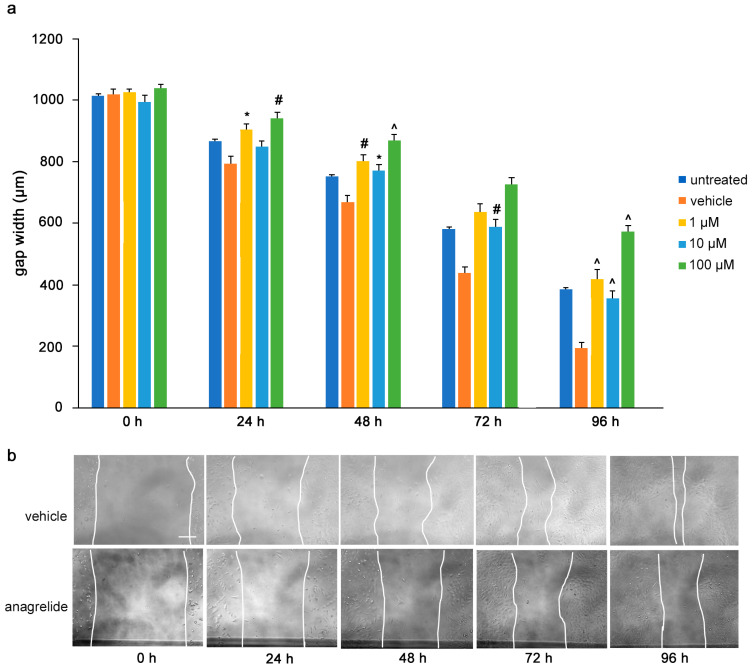
Anagrelide inhibits migration of U251 cells. U251 cells were seeded in 96-well plates. After 24 h, monolayers were scratched, immediately imaged, and then treated with different concentrations of anagrelide (1, 10 and 100 μM). Cells were imaged every 24 h up to 96 h. (**a**) Graph represents the gap width (μm) of untreated cells, vehicle-treated cells and cells treated with vehicle or 100 μM anagrelide. Two images were taken per well and values for each condition were averaged. Error bars show mean + SEM from 5 independent experiments. * *p* < 0.05, # *p* < 0.01, ^ *p* < 0.001 as determined by one-way ANOVA with Tukey’s post hoc test. (**b**) Representative images of anagrelide-treated and vehicle-treated cells. Scale bar for all images, 200 μm in panel for vehicle-treated cells.

**Figure 4:**
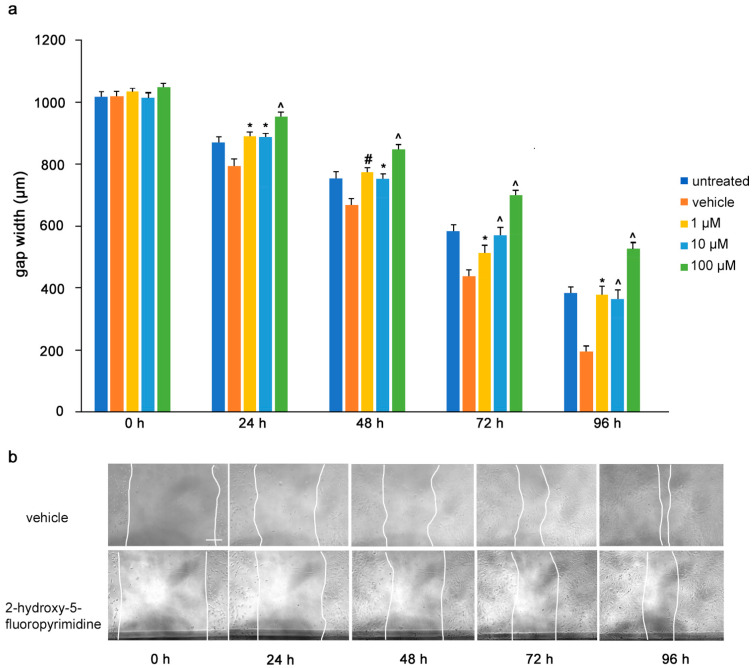
2-Hydroxy-5-fluoropyrimidine inhibits migration of U251 cells. U251 cells were seeded in 96-well plates. After 24 h, monolayers were scratched, immediately imaged, and then treated with different concentrations of 2-hydroxy-5-fluoropyrimidine (1, 10 and 100 μM). Cells were imaged every 24 h up to 96 h. (**a**) Graph represents the gap width (μm) of untreated cells and cells treated with vehicle or 2-hydroxy-5-fluoropyramidine. Two images were taken per well and values for each condition were averaged. Error bars show mean + SEM from 5 independent experiments. * *p* < 0.05, # *p* < 0.01, ^ *p* < 0.001 as determined by one-way ANOVA with Tukey’s post hoc test. (**b**) Representative images of 100 μM 2-hydroxy-5-fluoropyrimidine-treated and vehicle-treated cells. Scale bar for all images, 200 μm in panel vehicle, 0 h.

**Figure 5:**
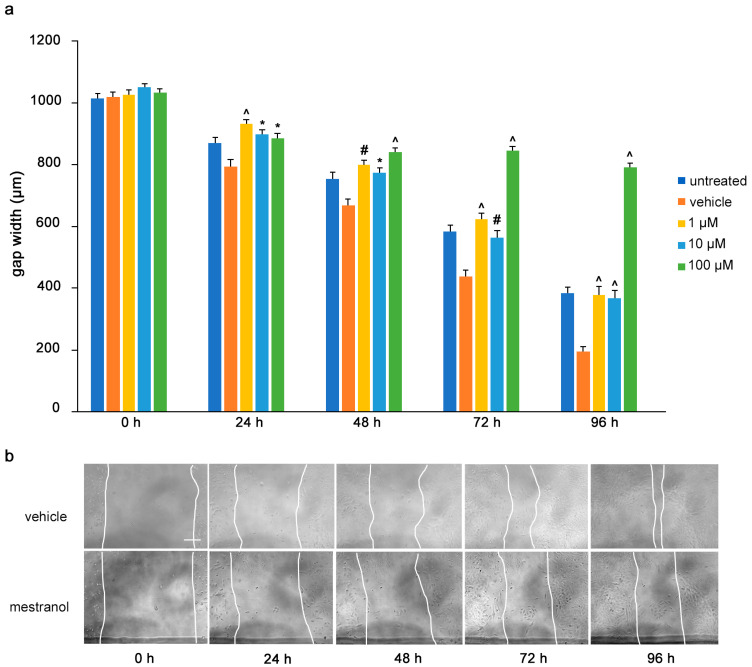
Mestranol inhibits migration of U251 cells. U251 cells were seeded in 96-well plates. After 24 h, monolayers were scratched, immediately imaged, and then treated with different concentrations of mestranol (1, 10 and 100 μM). Cells were imaged every 24 h up to 96 h. (**a**) Graph shows the gap width (μm) of untreated cells, vehicle-treated cells and cells treated with vehicle or mestranol. Two images were taken per well and values for each condition were averaged. Error bars show mean + SEM from 5 independent experiments. * *p* < 0.05, # *p* < 0.01, ^ *p* < 0.001 as determined by one-way ANOVA with Tukey’s post hoc test. (**b**) Representative images of 100 μM mestranol- and vehicle-treated cells. Scale bar for all images, 200 μm in panel vehicle, 0 h.

To determine the influence of the mimetics on tumor cell migrations in vitro, a monolayer of U251 cells was wounded by scratching and the gap width was measured. The cells were then treated with the L1 antagonistic mimetics at different concentrations (1–100 µM) and the effect of the compounds on scratch closure was monitored by imaging up to 96 h, with images taken every 24 h. At 72 and 96 h, the anagrelide-treated cells showed larger gap widths, whereas the vehicle control cells showed smaller gap widths at all concentrations tested (Figure 3a,b). Interestingly, treatment with 2-hydroxy-5-fluoropyrimidine resulted in a larger gap width already after 24 h at all concentrations tested compared to the vehicle control-treated cells (Figure 4a,b). Mestranol inhibited cell migration at all concentrations already after 24 h, but maximally at the higher concentration of 100 μM at 72 and 96 h (Figure 5a,b). These results indicate that the mimetics inhibit U251 cell migration at a concentration as low as 1 μM.

We also confirmed these findings in another glioblastoma cell line, A172, where, however, higher concentrations of the L1 mimetics were needed to observe significantly reduced migration (Supplementary Figure S1).

### 3.4. Inhibition of Migration by Mimetics Is L1 Dependent

**Figure 6:**
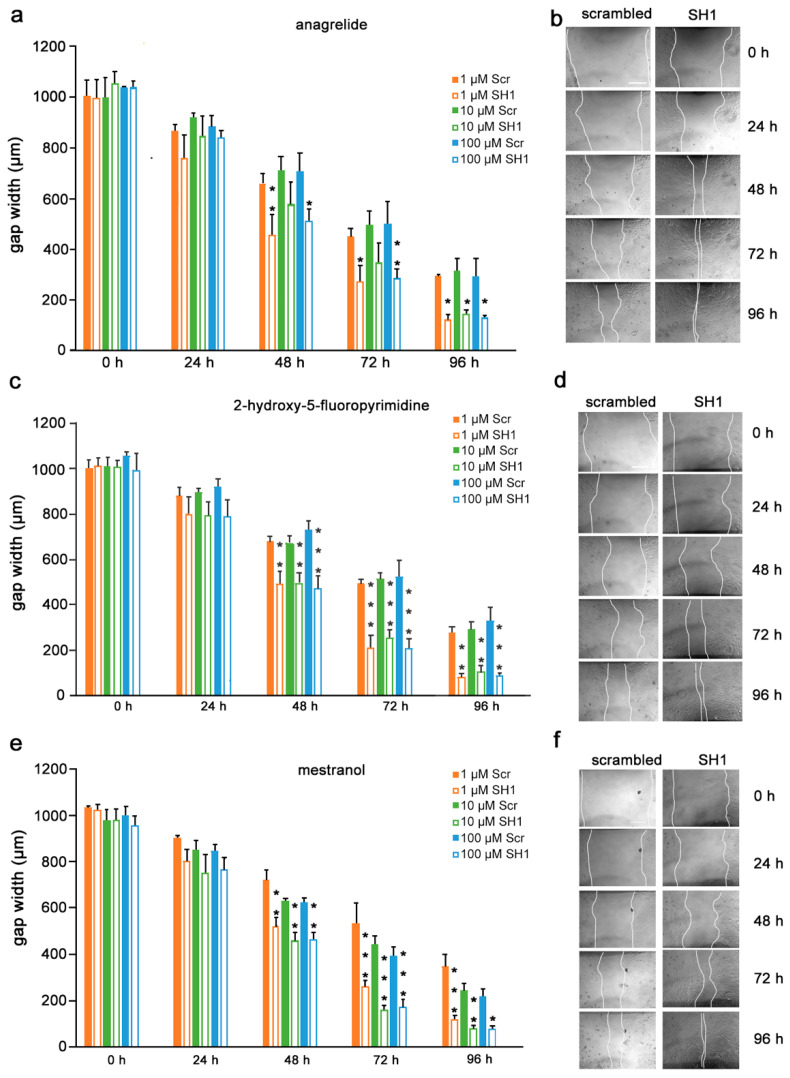
Mimetics do not inhibit migration of OVCAR3 cells after L1 knockdown. OVCAR3-scrambled and OVCAR3-SH1 cells were seeded in 96-well plates. After 24 h, monolayers were scratched, immediately imaged, and then treated with different concentrations of (**a**) anagrelide (**c**) 2-hydroxy 5-fluoropyramidine or (**e**) mestranol (1, 10 and 100 μM). Cells were imaged every 24 h up to 96 h. Migration of cells treated with mimetics is not inhibited compared to scrambled shRNA-treated cells from 48 h to 96 h. Data show mean + SEM. * *p*< 0.05, ** *p* < 0.01 and *** *p* < 0.001 difference to the respective scrambled shRNA (Scr) control, as determined by one-way ANOVA with Tukey’s post hoc test. (**b**,**d**,**f**) Representative images of OVCAR3 scrambled and SH1 cells treated with 100 μM mimetics. Scale bar for all images, 200 μm in panel (**a**) scrambled.

**Figure 7:**
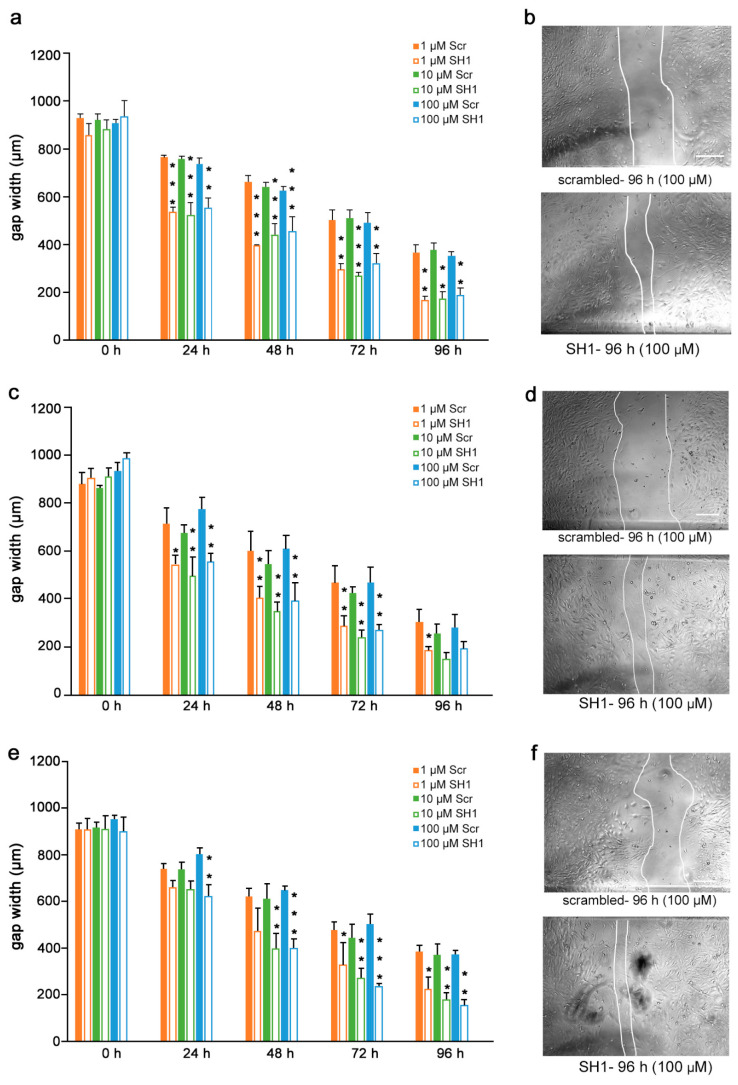
Mimetics do not inhibit migration of U251 cells after shRNA-mediated knock-down using L1 shRNA-SH1. U251-scrambled and U251-SH1 cells were seeded in 96-well plates. After 24 h, monolayers were scratched, immediately imaged, and then treated with different concentrations of (**a**) anagrelide (**c**) 2-hydroxy 5-fluoropyramidine and (**e**) mestranol (1, 10 and 100 μM). Cells were imaged every 24 h up to 96 h. Migration of cells treated with mimetics is not inhibited compared to scrambled shRNA-treated cells from 48 h to 96 h. Data show mean + SEM. * *p*< 0.05, ** *p* < 0.01 and *** *p* < 0.001 difference to the respective scrambled shRNA (Scr) control, as determined by one-way ANOVA with Tukey’s post hoc test. (**b**,**d**,**f**) Representative images of U251 scrambled and SH1 cells treated with 100 μM mimetics. Scale bar for all images, 150 μm as in upper panel scrambled.

**Figure 8:**
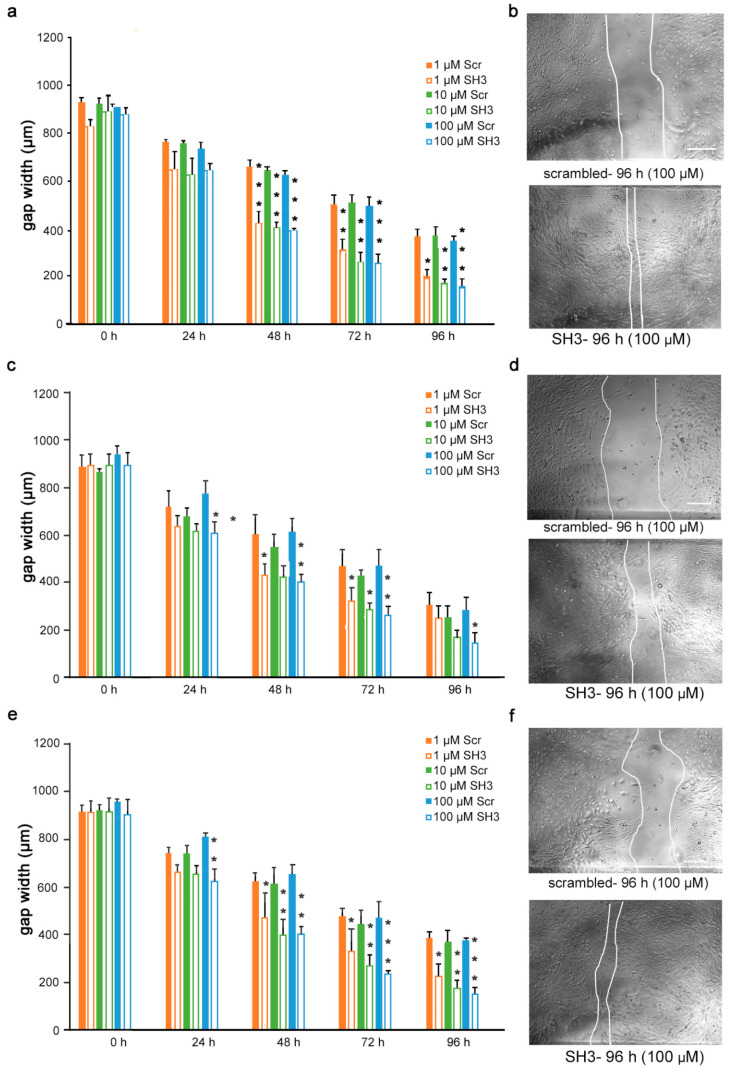
Mimetics do not inhibit migration of U251 cells after shRNA-mediated L1 knock-down using L1 shRNA-SH3. U251-scrambled and U251-SH3 cells were seeded in 96-well plates. After 24 h, monolayers were scratched, immediately imaged, and then treated with different concentrations of (**a**) anagrelide (**c**) 2-hydroxy 5-fluoropyramidine or (**e**) mestranol (1, 10, 100 μM). Cells were imaged every 24 h up to 96 h. Migration of cells treated with mimetics is not inhibited compared to scrambled shRNA-treated cells from 48 h to 96 h. Data show mean + SEM. * *p*< 0.05, ** *p* < 0.01 and *** *p* < 0.001 difference to the respective scrambled shRNA (Scr) control, as determined by one-way ANOVA with Tukey’s post hoc test. (**b**,**d**,**f**) Representative images of U251 scrambled and SH3 cells treated with 100 μM mimetics. Scale bar for all images, 150 μm as in upper panel scrambled.

Given the findings that the mimetics competitively bind to L1 and inhibit migration of U251 cells, we next determined if the inhibition is L1-dependent. To this end, U251 cells were stably transduced with lentiviral vectors carrying two different shRNAs targeting two different regions of the L1 genome. Moreover, to test whether the L1 mimetics would inhibit migration in a different cell type, we applied the same approach to OVCAR3, an ovarian carcinoma cell line. The expression of L1 after treatment with SH1 and SH3 and the respective knock-down efficiency of the shRNAs compared to the respective scrambled controls is shown in Supplementary Figure S2 (U251) and [31] (OVCAR3). OVCAR3-SH1 (Figure 6), U251-SH1 (Figure 7) and U251-SH3 (Figure 8) and the respective scrambled control cells were treated with the L1 mimetics at different concentrations (1–100 µM) and the effect of the compounds on scratch closure was monitored by imaging up to 96 h, with images taken every 24 h. In both cell lines, the mimetics did not inhibit the migration of cells treated with L1 shRNAs compared to the cells treated with scrambled shRNA. With OVCAR3 cells treated with anagrelide (Figure 6a), 2-hydroxy-5-fluoropyrimidine (Figure 6c) and mestranol (Figure 6e), the gap width closure was seen from 48 h onward at all concentrations tested. Similar results were obtained with U251 cells. For these cells, two different shRNAs SH1 (Figure 7) and SH3 (Figure 8) were used to reduce L1 expression. In both L1-silenced cell types, all the mimetics were less efficient in inhibiting migration compared to the cells transduced with the scrambled construct at all concentrations tested. Taken together, these results show that the mimetics inhibit migration in a L1-dependent manner. 

## 4. Discussion

Tumor therapy has given encouraging results with antibodies directed against L1 [34,35,36,37]. However, since tissue penetration, crossing of the blood–brain barrier, half-life and stability of antibodies are not optimal for therapy, we searched for small molecule antagonists of L1 that should allow favorable translation into therapeutic drugs. We identified three compounds that inhibited the binding of monoclonal antibody 324 to L1 and bound to L1, as would be expected for compounds triggering L1 functions in a homophilic manner. Antibody 324 binds to the second immunoglobulin-like domain of L1, which is different from the binding of monoclonal antibody 557, which binds to the third fibronectin type III homologous repeat and was used to identify L1 agonistic compounds [18]. The second immunoglobulin-like domain is interesting from another point of view: ethanol, but not methanol nor propranolol binds to it, and if ethanol is consumed at mM concentrations by the mother in the first trimester of pregnancy, the child is at risk of developing fetal alcohol syndrome, which shows many features of L1 syndrome (for a review, see [1]). 

The identified L1 antagonistic mimetics are components of drugs that are currently used for treatment of different diseases and are thus FDA-approved. Anagrelide is a blood thinner used to treat thrombocythemia by reducing the platelet count. It prevents megakaryocyte maturation in the bone marrow by reducing the platelet count without interfering with other progenitor cell lines in the bone marrow [38]. Megakaryocytes are inhibited to mature by anagrelide, resulting in decreased levels of transcription factors GATA-1, FLI-1, and NF-E2, possibly by being an upstream regulator of both GATA-1 and FOG-1 [39,40]. Anagrelide also decreases the platelet count by inhibiting pro-platelet formation. Pro-platelets are extrusions from mature megakaryocytes that extend into the bone marrow sinusoidal lumen and give rise to platelets at the tips of their protrusions [40]. 

2-Hydroxy-5-fluoropyrimidine is converted to 5-fluorouracil by hepatic aldehyde oxidase, a process which is inhibited by compounds such as potassium cyanide, menadione, and diethylstilbestrol [41]. 5-Fluorouracil inhibits metabolic processes in cells and is incorporated into the RNA of tumor cells more abundantly than into non-tumor cells because of the tendency of tumor cells to upregulate protein synthesis compared to non-tumor cells. The incorporation of 5-fluorouracil into RNA renders RNA non-functional, thereby reducing vital cell functions. Furthermore, 5-fluorouracil inhibits thymidylate synthase, an enzyme that converts deoxyuridine monophosphate to deoxythymidine monophosphate [42]. Deoxythymidine monophosphate is a precursor of deoxythymidine triphosphate, a nucleotide necessary for DNA synthesis. 5-Fluorouracil irreversibly inhibits thymidylate synthase by causing substrates to bind covalently to the enzyme [43]. Interestingly, 5-fluorouracil is most effective in colorectal cancer, a cancer where L1 is overexpressed, especially at the invasive front of the tumor [44]. This finding could be interpreted by the view that 5-fluorouracil inhibits tumor cell migration by its L1 antagonistic activity, and in addition to disturbing DNA and RNA synthesis.

Mestranol is an estrogen that continues to be used as a contraceptive. Although estrogen is naturally found in the human body, consistent exposure to exogenous estrogen has been shown to increase the risk of developing various cancers [45]. Reports on the effect of mestranol on tumorigenesis have generated conflicting data. With regard to hepatocarcinoma, the incidence of tumorigenesis is increased with increasing mestranol concentrations administered to rats [46]. However, a “consistent gender disproportion” in hepatocarcinoma incidence in humans has suggested that premenopausal women are more protected from hepatocarcinoma because of high blood estrogen levels, and premenopausal women experience better recoveries from hepatocarcinoma [47]. These reports are interesting in that different isoforms of the estrogen receptor may be expressed in normal cells compared to cancerous hepatocytes. Such a difference suggests that estrogen administration to cancerous hepatocytes may increase tumor aggression because of estrogen receptor overexpression or maladaptation, not because of harmful effects of estrogen itself [47]. In fact, the administration of norethynodrel-mestranol has been shown to decrease mammary gland carcinogenesis in rats by altering the gland structure to augment protection against carcinogenesis [48]. We would like to speculate that the different types of mestranol may derive from its possibly dual activities: one would be as a compound that binds to L1 at the cell surface in a homophile manner, resulting in a broad spectrum of cellular consequences from the cell surface to signal transducers, effects on mitochondria and transcription in the nucleus. Mestranol’s other functions pertain to its cognate effects on estrogen receptors. 

How these at first sight different modes of actions would affect each other and how they might merge in possibly even epigenetic situations will need to be addressed in complex sets of future experiments. The latter will need to include other types of L1-positive tumor cell lines, such as different glioblastoma and neuroblastoma cells, as well as patient-derived tumors where the metastatic potential correlates with L1 expression. We need to emphasize in this context that in vivo experiments will have to be performed to evaluate the effects of the three mimetics on tumors of mouse and human origins, with the latter requiring the implantation of tumors into immune-deficient recipients. We also need to note that the discovered compounds impact non-tumor-related functions. These would have to be taken into account as possible contra-indicative outcomes. In the case of mestranol, for instance, effects on estrogen receptors need to be considered. It is also worth considering that the concentrations needed to reduce tumor cell migration may be different from those affecting other cell types. Investigations on these questions are planned for the future.

## 5. Conclusions

All L1 antagonistic drugs reported here have known toxicological and pharmacokinetic profiles, and their repurposing would thus reduce the need for the establishment of a fully new therapeutic profile. Our novel findings that these compounds act as L1 antagonists raises hopes that they may be useful for therapy of a wide variety of cancers. 

## Data Availability

Not applicable.

## Supplementary Materials

The following are available online at https://www.mdpi.com/article/10.3390/biom12030439/s1, Figure S1: Migration assay performed in A172 human glioblastoma cell line; Figure S2: Western blots show shRNA-mediated L1 reduction in U251 cells.
