# Supplementary material for: Antagonistic L1 Adhesion Molecule Mimetic Compounds Inhibit Glioblastoma Cell Migration In Vitro
Source: Biomolecules. 2022 Mar 12;12(3):439. doi: 10.3390/biom12030439 (PMC8946856; doi:10.3390/biom12030439)
Supplement: Supplementary file 1 [file biomolecules-12-00439-s001.zip › biomolecules-1505524-supplementary.pdf]

## Supplementary materials and methods and figure legends:

### ELISA with L1-Fc:

Determination of antibody 324 concentration-dependent binding to mouse L1-Fc was conducted via ELISA as described [29] with some modifications. To titrate the binding of antibody, L1-Fc (2.5 µg/ml, 25 µl/well) was immobilized in 96-well flat bottom microtiter plates with medium binding surface capacity (Cat# 9017; Corning). The plate was centrifuged for 1 min at 200 g and then allowed to stand for 24 h at 4°C. After aspiration of the supernatant, the wells were treated with blocking solution (5% BSA in PBS, pH 7.3) for 1 h at room temperature on a shaker at 250 rpm. The blocking solution was then discarded and 25 µl of different concentrations of antibody 324 solution (0, 0.2, 1.0, 2.5 and 5.0 µg/ml) were added to the wells in duplicates. Plates were then incubated for 30 min at room temperature on the shaker, washed three times with PBS, followed by addition of donkey anti-rat HRP-coupled secondary antibody (1:400, 25 µl/well). Subsequently, wells were washed three times with PBS, after which the peroxidase substrate (1 mg/ml o-phenylenediamine dihydrochloride dissolved in 50 mM citric acid, 50 mM sodium phosphate and 0.03% hydrogen peroxide) was added (25 µl/well). After optimal signal development, the reaction was stopped by adding a 2.5 M sulfuric acid. Absorbance (optical density - OD) was determined at wavelengths 450 and 570 nm using an ELISA reader (EL800; BioTek Instruments, Winooski, VT, USA).

### Supplementary Figure S1:

**Materials and methods.** A172 cells (human glioblastoma cell line) were obtained from Istituto Zooprofilattico di Brescia and authenticated by short tandem repeat profiling. Cells were cultured in DMEM, 10% fetal bovine serum, penicillin/streptomycin. The migration assay was performed as described for U251 cells, and images were acquired every 12 hours.

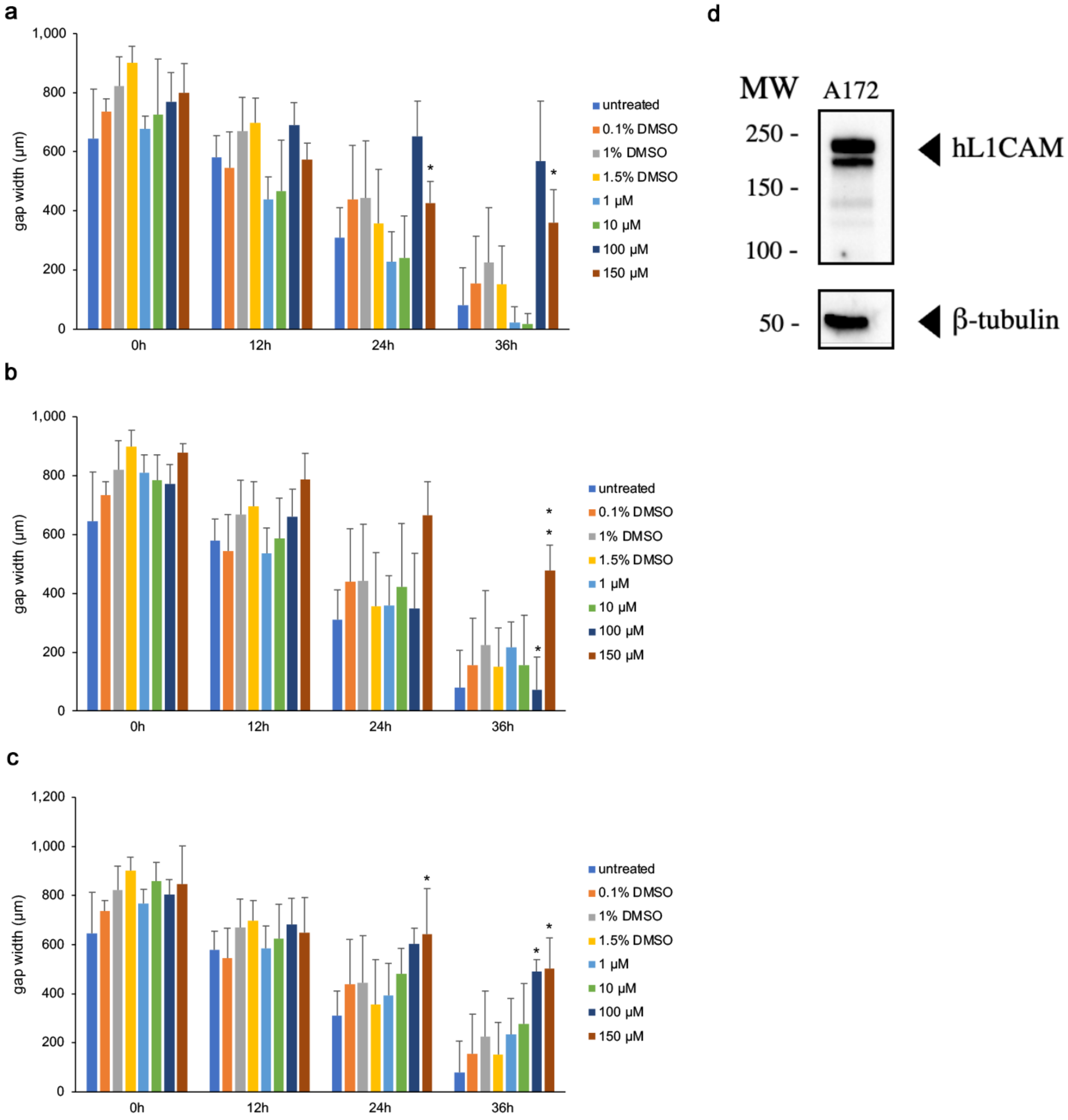


**Figure S1.** A172 cells were seeded in 96-well plates. After 24 h, monolayers were scratched, immediately imaged, and then treated with different concentrations of **(a)** anagrelide **(b)** 2-hydroxy 5-fluoropyramidine or **(c)** mestranol (1, 10, 100, 150 µM). Cells were imaged every 12 h up to 36 h. Migration of cells treated with mimetics is inhibited if compared to the vehicle at 36h. Data show mean + SEM. * $p < 0.05$, ** $p < 0.01$ and *** $p < 0.001$ difference to the respective vehicle control, as determined by one-way ANOVA with Tukey's post-hoc test. **(d)** Expression of L1 in A172 cells was assessed by Western blot using antibody against human L1 (1:200). β-tubulin was used for normalization.

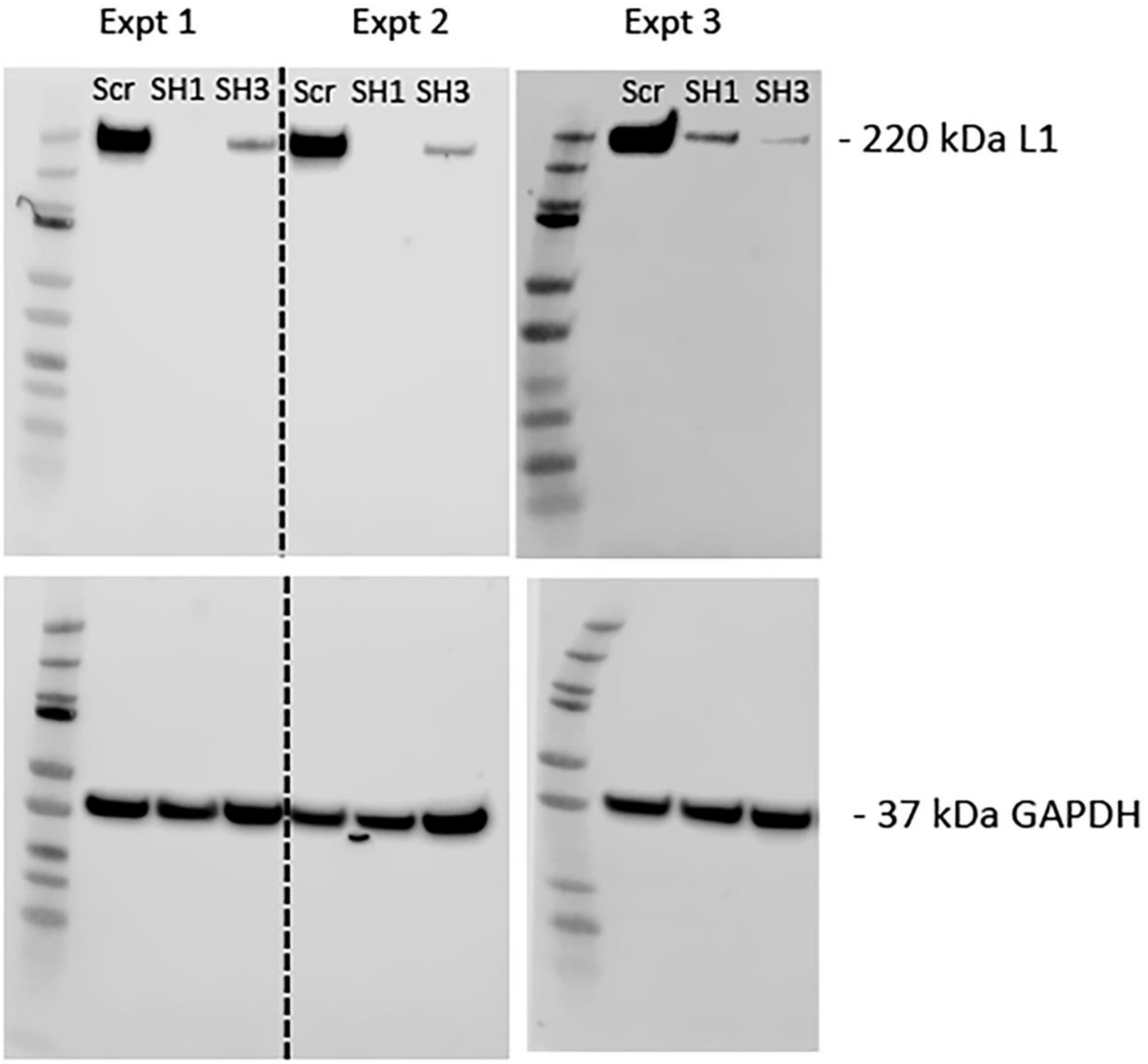


**Figure S2.** Western blots show shRNA-mediated L1 reduction in U251 cells. Expression of L1 in U251 cells treated with L1 viral construct (Scr), constructs SH1 (L1 shRNA 1) and SH3 (L1 shRNA 3) was assessed by three independent Western blots experiments using antibody against human L1 (1:200). GAPDH was used for normalization. The first two independent experiments (Expt 1 and Expt 2) are shown in one blot; separation is shown by a dotted line. The third experiment (Expt 3) is shown in an individual blot.
